# Genetic Variability in Susceptibility to Occupational Respiratory Sensitization

**DOI:** 10.1155/2011/346719

**Published:** 2011-06-12

**Authors:** Berran Yucesoy, Victor J. Johnson

**Affiliations:** Toxicology and Molecular Biology Branch, Health Effects Laboratory Division, National Institute for Occupational Safety and Health, Centers for Disease Control and Prevention, Morgantown, WV 26505, USA

## Abstract

Respiratory sensitization can be caused by a variety of substances at workplaces, and the health and economic burden linked to allergic respiratory diseases continues to increase. Although the main factors that affect the onset of the symptoms are the types and intensity of allergen exposure, there is a wide range of interindividual variation in susceptibility to occupational/environmental sensitizers. A number of gene variants have been reported to be associated with various occupational allergic respiratory diseases. Examples of genes include, but are not limited to, genes involved in immune/inflammatory regulation, antioxidant defenses, and fibrotic processes. Most of these variants act in combination with other genes and environmental factors to modify disease progression, severity, or resolution after exposure to allergens. Therefore, understanding the role of genetic variability and the interaction between genetic and environmental/occupational factors provides new insights into disease etiology and may lead to the development of novel preventive and therapeutic strategies. This paper will focus on the current state of knowledge regarding genetic influences on allergic respiratory diseases, with specific emphasis on diisocyanate-induced asthma and chronic beryllium disease.

## 1. Introduction

Workplace allergens can be categorized as either high or low molecular weight allergens. Low molecular weight (LMW) allergens such as diisocyanates, acid anhydrides, and metallic salts are reactive chemicals with molecular weight less than 1000 kD. They act as haptens and can cause sensitization that may or may not be associated with specific immunoglobulin E (IgE). While some LMW agents such as acid anhydrides, platinum salts, and persulfates stimulate IgE antibodies, many others including isocyanates and glutaraldehyde rarely cause IgE-mediated sensitization [1]. On the other hand, high molecular weight (HMW) protein-derived agents (e.g., proteases, flour, and laboratory animal allergens) cause allergic sensitization through mechanisms mediated by IgE [2]. Early detection of sensitization is very important since sensitized individuals can have life-threatening reactions to future exposures even years after the cessation of exposure. Although the risk of sensitization for individuals with underlying atopy is higher for some exposures (particularly IgE-mediated responses), high prevalence (around 20%) of atopy in the general population indicates that atopy alone is not the determining factor [3]. Although the main factors that affect the onset of the symptoms are the types, duration, and intensity of allergen exposure, host genetic factors can modulate how individuals interact with these agents and induce a shift in the dose-response relationship [4]. Recent genetic epidemiology research focused on common gene variants and identified a number of genetic associations and gene-environment interactions for allergic respiratory diseases. Understanding gene-environment interactions is especially important to improve occupational and public health since environmental/occupational factors that influence genetic risk are modifiable. In this respect, the results of molecular epidemiology studies have the potential to be used in risk evaluation and to help determine more accurate safe occupational exposure levels, thereby contributing to improved protection of workers at high risk. This paper will summarize the contribution of genetic variability to two important occupational respiratory diseases, diisocyanate-induced asthma (DA) and chronic beryllium disease (CBD).

## 2. Occupational Asthma Caused by Diisocyanates

Among LMW substances, the diisocyanates are the most frequently reported cause of respiratory sensitization in the workplace. These agents are widely used in polymerization reactions for manufacturing surface coatings, varnishes, paints, urethane foams, insulation, and adhesives. Workers in these industries and workers that use these end products may be influenced by potential adverse health effects of such chemicals. National Occupational Exposure Survey database, National Institute for Occupational Safety and Health (NIOSH), showed that at least 280,000 workers were potentially exposed to some form of isocyanates in the United States alone [5]. Isocyanates are the leading cause of occupational asthma (OA), estimated to cause asthma in 5–10% of chronically exposed workers [6–8]. Despite improved industrial hygiene efforts, new cases of OA continue to occur [9, 10]. The most common isomers used in industry are: the aliphatic agent 1,6-hexamethylene diisocyanate (HDI), used principally as a hardener in spray paints, 4,4-diphenylmethane diisocyanate (MDI), and toluene diisocyanate (TDI). Early diagnosis of OA leads to favorable clinical outcomes (i.e., less risk of chronic and severe asthma) if affected workers are promptly recognized and removed from harmful exposure [9, 11]. In addition to early case detection, it is also important to more closely monitor the most susceptible workers at a preclinical stage. 

Genetic epidemiologic studies have identified a number of susceptibility markers for a variety of asthma phenotypes including OA. Most of these genetic studies were hindered by difficulty in defining asthma, a complex phenotype representing allergic and nonallergic types. This has led to selection of intermediate or quantifiable phenotypes (e.g., airway hyperresponsiveness, lung function, and serum IgE levels) in some association studies. On the other hand, OA is a unique model in that the phenotype can be defined accurately by specific inhalation challenge (SIC) testing often considered the gold standard for diagnosing OA [12]. For this reason, OA is an excellent model for studying gene-environment interactions since the causal agent can be identified with SIC and the lag period between initial exposure and onset of sensitization and clinical symptoms can be followed [13]. 

Given its immune-inflammatory nature, OA phenotypes are likely associated with specific variants of immune-/inflammatory-related genes. Linkage studies have suggested a variety of candidate genes for asthma and related phenotypes in chromosomal regions 2q14-q32, 5q31-q33, 6p24-p21, 7p15-p14, 11q13-q21, 12q21-q24, 13q12-q14, and 20p13 [14–21]. In particular, variants of interleukin (IL)-4, IL-4RA, IL-13, *β*-adrenergic receptor (*β-*AR), tumor necrosis factor (TNF)-*α*, human leukocyte antigen (HLA)-DRB1, DQB1, the *β-*subunit of the high-affinity IgE receptor (FceRI), CD14, a disintegrin and a metalloproteinase 33 (ADAM33) genes have been consistently associated with asthma-related phenotypes in independent studies [22, 23]. As in other forms of asthma, inflammatory changes and allergen-specific T-lymphocytes are found in the airways of many patients with OA, along with eosinophils, cytokines, and serum IgE antibodies [24–26]. Thus, similar genetic associations as in immune-mediated asthma might be expected to occur in OA. Although a number of genetic association studies have been conducted on individuals with allergic asthma from environmental causes, there are only limited studies on OA. 

The Human Leukocyte Antigen (HLA) class II molecules play a role in the presentation of intracellularly processed peptides to CD4+ T-helper cells. HLA class II molecules are highly polymorphic and the variations in their protein structure may determine the specific epitopes presented to T cells. Therefore, HLA class II molecules are also plausible candidates for controlling specific immunological responses to allergens. Genetic studies investigating the immunopathogenesis of OA have focused on HLA Class II alleles. Bignon et al. demonstrated that HLA DQB1*0503 and the allelic combination DQB1*0201/0301 were associated with susceptibility to DA [27] whereas the DQB1*0501 allele and the DQA1*0101-DQB1*0501-DR1 haplotype were considered protective. Subsequently, Mapp et al. confirmed the association with HLA-DQB1*0503 and reported that the DQA1*0104 allele was increased in DA compared with asymptomatic exposed workers. They also showed that “protective” alleles, HLA-DQB1*0501 and DQA1*0101, were increased in asymptomatic exposed workers versus those with DA [19]. In another study, a significantly higher proportion of subjects with DA were found to express the HLA-DQB1*0503-associated aspartic acid at residue 57 [28]. HLA associations with DA were also investigated in a population of Asian workers exposed to diisocyanates but the associations found in European workers were not entirely replicated. The HLA haplotypes DRB1*15-DPB1*05 and HLA DRB1*1501-DQB1*0602-DPB1*0501 were reported as a susceptibility marker for the development of TDI-induced asthma in Koreans [29, 30]. Bernstein et al., investigated association between known SNPs in immune response genes (IL-4R*α*, IL-13, and CD14) and DA in a group of exposed workers undergoing SIC testing. The results demonstrated increased frequencies of IL-4RA I50V allele and combinatorial genotypes of IL4RA (I50V), IL-13 (R110Q), and CD14 (C159T) in HDI-exposed workers suggesting an exposure-specific interaction [31]. These finding supported the notion that immune mechanisms play an important role in the pathogenesis of DA. 

Since isocyanates are known to cause oxidative injury to respiratory epithelial cells, variations within antioxidant defense genes have been examined in workers with DA [32]. Glutathione, a major antioxidant protein found in the bronchial lining fluid and in respiratory epithelial cells, is likely to serve a protective function by binding with free isocyanate molecules and, thereby, preventing damage to respiratory epithelial cells or intracellular binding to respiratory epithelial proteins or proteins in the bronchial lumen [33]. Piirilä et al. examined polymorphisms of the glutathione S-transferase (GST) genes (GSTM1, GSTM3, GSTP1, and GSTT1) in workers with DA. GSTM1 null genotype was associated with a 1.89-fold risk of DA. Subjects with GSTM1 null and GSTM3 AA genotypes developed late reaction in the specific bronchial provocation test with diisocyanates, individually or in combination [34]. Later, Mapp et al. assessed the GSTP1 gene in TDI-exposed asymptomatic and asthmatic workers [18]. The frequency of the GSTP1 Ile105Val Val/Val genotype was lower in subjects with DA, and was significantly lower among subjects with airway hyperresponsiveness. In another study, the N-acetyltransferase (NAT1) slow acetylator genotype was associated with a 2.5-fold risk of OA among diisocyanate-exposed workers. Interestingly, a far greater 7.77-fold risk of OA was reported among workers exposed to TDI, suggesting an exposure-specific association. In addition, a gene-gene interactive effect was identified in diisocyanate-exposed workers with the combined NAT1 or NAT2 slow acetylator genotypes and GSTM1 null genotype [35]. Broberg et al. investigated the influence of variants in TDI-metabolizing genes on the associations between TDI in air (2,4-TDI and 2,6-TDI) and its metabolites toluene diamine (2,4-TDA and 2,6-TDA) in plasma and urine. Their results showed that the GSTP1 Ile105Val variant modifies the association between 2,4-TDA in plasma and in urine, supporting the importance of GST system for the metabolism of TDI [36]. Based on the role of neurogenic inflammation in TDI-induced airway hyperresponsiveness, the association between neurokinin-2 receptor (NK2R) gene polymorphisms and TDI-induced asthma was investigated in a Korean population. An association was found between the NK2R 7853GG genotype and increased serum VEGF levels, suggesting that NK2R variants may modulate the airway inflammation conferred by VEGF [37]. Another Korean study investigated the possible role of *β*2-adrenergic receptor gene (ADRB2) polymorphisms in TDI-induced asthma. The Arg16Gly A>G, Leu134Leu G>A, and Arg175Arg C>A SNPs and haplotype [TTACGC] were found to be associated with specific IgE sensitization in TDI-exposed workers [38]. In another study, genome-wide association was performed to identify susceptibility alleles associated with asthma induced by TDI. The results showed significant association between genetic polymorphisms (rs10762058, rs7088181, rs4378283, and rs1786929) of catenin *α*3 (CTNNA3) and susceptibility to TDI-induced asthma [39]. The CTNNA3 variants have been suggested to influence TDI-induced asthma risk by increasing epithelial damage and airway inflammation.

## 3. Chronic Beryllium Disease

Chronic beryllium disease (CBD) is a serious granulomatous lung disease caused by beryllium (Be) exposure in the workplace. CBD continues to occur in industries where Be is manufactured and processed such as aerospace, nuclear, automotive, and electronics. NIOSH estimated that up to 134,000 workers in the United States were exposed to beryllium [40]. Be exposure leads to a cell-mediated hypersensitivity (delayed, type IV) reaction in which Be haptenates native proteins leading to the production of the specific allergen [41]. It is known that accumulation of Be-specific CD4(+) T cells and persistent lung inflammation play a key role in the immunopathogenesis of CBD. Prior to the development of CBD, many exposed workers become sensitized and many of those eventually develop CBD. Approximately 50% of sensitized individuals have CBD at initial clinical evaluation [42]. Be-specific T-cell proliferative responses are detected in the blood of exposed workers using the Be lymphocyte proliferation test (BeLPT) [43, 44]. The BeLPT has been shown to identify approximately 70 to 94% of cases of BeS and CBD and widely used in screening and surveillance of Be-exposed workers [45–47]. Epidemiological studies showed the prevalence rates of BeS and CBD to be between 5–21% and 3–21% among beryllium workers, respectively [48, 49]. The pathologic progression from BeS to CBD is not well understood warranting further research into the pathophysiological mechanisms and susceptibility markers of BeS and CBD. Such efforts will be important for early detection and disease prevention in Be-exposed workers. 

A number of molecular epidemiology studies showed that the presence of glutamic acid in position 69 of the B1 chain of the HLA-DPB1 molecule confers an increased risk for both BeS and CBD [41, 50–54]. The HLA-DPB1Glu69 frequency was reported to be between 39–90% in sensitized workers and 53–97% in workers with CBD as compared to 19–48% in nonsensitized workers [41, 50–52, 55–61]. Importantly, studies have demonstrated a dose-dependent effect of HLA-DPB1Glu69 alleles suggesting that Glu69 is a potential marker of disease severity in addition to overall disease risk [41]. Although HLA-DPB1Glu69 is more frequent in individuals with BeS and CBD (73–95%), 30–40% of exposed workers carrying HLA-DPB1Glu69 do not develop CBD or BeS [41, 50, 62]. This suggests that other host and environmental factors likely play key roles in the pathogenesis of CBD. Studies investigating the interaction between the HLA-DPB1 Glu69 and Be exposure showed independent and additive effects of Glu69 carriage and Be exposure in the development of BeS and CBD [58, 63]. 

Rossman et al. reported that HLA-DQB1Gly86 and HLA-DRB1Ser11 alleles occurred more often in individuals with CBD [55]. Maier et al. found that HLA-DRB1*01 and DQB1*05 alleles were less frequent in workers with CBD. They also reported that DRB1*13 and DQB1*06 were associated with CBD in the absence of Glu69 [41]. A recent study showed that the DR*β*E71 allele is a risk factor for both CBD and BeS in the absence of Glu69 and highlighted the importance of interactions between peptides and T cells in the development of CBD [61]. Chemically specific Be-protein interactions were also investigated using a computational approach. Glu69 alleloforms with the greatest negative surface charge were found to confer the highest risk for CBD and irrespective of allele, equal risk for BeS [64]. Current HLA research includes investigating whether the risk is associated with any or only certain Glu69 alleles or allelic combinations. 

Non-HLA genetic studies also identified some significant associations. Sato et al. investigated eight SNPs within CCR5 gene that is implicated in the chemotaxis and activation of leukocyte subsets. The results showed that CCR5-5663 and -3458 variants were associated with worsening pulmonary function over time in CBD [65]. The -509C and codon 10T variants of the transforming growth factor-*β*1 (TGF*β*) gene, a cytokine with a major role in the fibrotic/Th1 response, were found to be associated with more severe granulomatous disease in CBD [66]. Since glutathione has been reported to be increased in CBD, genetic variants of the glutamate cysteine ligase (GCL), a rate-limiting enzyme in GSH synthesis, were investigated. GCL consists of a catalytic subunit (GCLC) and modifier subunit (GCLM). GCLC trinucleotide repeat polymorphism (7/7 genotype) and the GCLM-588 SNP were found to be associated with altered susceptibility to CBD [67]. While Saltini et al. reported an association between the TNF*α*-308*02 variant and BeS and CBD, this result was not confirmed in a large population-based study [57, 68]. A recent study showed that IL-1*α*-1142, -3769, and -4697 variants were significantly associated with CBD compared to individuals with BeS or nonsensitized workers after adjusting for Glu69 status [69]. These results suggested that the formation of granulomas in CBD may require an independent inflammatory response controlled by genes unrelated to beryllium recognition.  Table 1 lists some examples of associations found for both DA and CBD.

## 4. Conclusions

Genetic association studies can provide more accurate information on the interindividual variability, thereby contributing to establishment of more accurate exposure limits in the workplace. These efforts, in a larger perspective, provide opportunities to effectively target engineering controls, personal protective equipment, and intervention strategies to protect the health of high-risk workers. With the advances in high-throughput technologies and computational methodologies, this information could be used in designing better predictive models to incorporate genetic variability into risk evaluation and improving the regulation and redefinition of acceptable exposure levels in the workplace. Success of such approaches depends on how molecular epidemiology studies overcome some of the current challenges. Despite the rapid growth of published associations, some of the genetic associations lack consistency across different studies. The inconsistency in results might be explained by the differences in study populations, phenotype characterization, exposure assessment, characterization of other environmental exposure (e.g., air pollution, smoking), intermediate phenotypes (e.g., airway hyperresponsiveness), statistical inconsistencies and other potentially modifiable risk factors such as lifestyle. For example, allele/carrier frequencies of the HLA-DPB1Glu69 ranged between 0.21/0.38 to 0.33/0.59 across different ethnic populations [70]. This emphasizes the importance of replication studies in independent populations with a different genetic background. Although the genetics of allergic respiratory diseases including DA and CBD have yet to be fully characterized, summarized discoveries hold promise for the identification of susceptibility profiles and characterization of gene-environment interactions. It is to be hoped that future genetic association studies with large, well-characterized populations through national and international collaborations will increase the understanding of the pathogenesis of these diseases and help identify novel therapeutic targets and preventative/educational strategies for better identification and management of occupational diseases.

## Figures and Tables

**Table 1:** Examples of genetic associations for DA and CBD.

Disease	Gene	Variation	RR; *P* value; OR (95% CI)	Reference
DA				

	HLA-DQB1	*0503	RR = 9.8, *P* < .04	[27]
	HLA-DQB1	*0501	RR = 0.14, *P* < .03	[27]
	HLA-DQA1	*0104	*P* = .008	[19]
	HLA-DRB1-DPB1	*15*05	*P* = .001	[29]
	GSTM1	Null	1.89 (1.01–3.52)	[34]
	NAT1	Slow acetylator	7.77 (1.18–51.6)	[35]
	IL4RA, IL-13 R, CD14	I50V-R110Q-C159T	6.4 (1.57–26.12)	[31]
	CTNNA3	rs1786929	*P* = .015	[39]

CBD				

	HLA-DPB1	Glu69	9.4 (5.4–16.6)	[50]
	HLA-DQB1	G86	*P* < .04	[55]
	HLA-DRB1	S11	*P* < .03	[55]
	CCR5	-3458	*P* < .0001	[65]
	TGF*β*1	-509	*P* = .01	[66]
	GCLC	TNR 7/7	0.28 (0.08–0.95)	[67]
	GCLM	-588 C/C	3.07 (1.00–9.37)	[67]
	IL-1A	-1142	3.02 (1.36–6.70)	[69]
		-3769	2.51 (1.21–5.19)	[69]
		-4697	2.56 (1.24–5.29)	[69]

RR: relative risk; OR: odds ratio.
